# Stellettin B induces apoptosis in human chronic myeloid leukemia cells via targeting PI3K and Stat5

**DOI:** 10.18632/oncotarget.15957

**Published:** 2017-03-07

**Authors:** Yali Chen, Qianxiang Zhou, Lei Zhang, Yuxu Zhong, Guanwei Fan, Zhe Zhang, Ran Wang, Meihua Jin, Yuling Qiu, Dexin Kong

**Affiliations:** ^1^ Department of Biopharmaceutical Sciences, Tianjin Key Laboratory on Technologies Enabling Development of Clinical Therapeutics and Diagnostics, School of Pharmacy, Tianjin Medical University, Tianjin 300070, China; ^2^ Research Center of Basic Medical Sciences, Tianjin Medical University, Tianjin 300070, China; ^3^ State Key Laboratory of Toxicology and Medical Countermeasures, Beijing Institute of Pharmacology and Toxicology, Beijing 100850, China; ^4^ Institute of Traditional Chinese Medicine Research, State Key Laboratory of Modern Chinese Medicine, Tianjin University of Traditional Chinese Medicine, Tianjin 300193, China

**Keywords:** stellettin B, K562, apoptosis, PI3K, combination

## Abstract

Novel agents are still urgently expected for therapy of chronic myeloid leukemia (CML). The *in vitro* anti-leukemia activity of Stellettin B (Stel B), a triterpenoid we isolated from marine sponge *Jaspis stellifera*, on human CML K562 and KU812 cells was recently investigated. Stel B inhibited K562 and KU812 cell proliferation with IC_50_ as 0.035 μM and 0.95 μM respectively. While no obvious cell cycle arrest was observed, apoptosis was induced in K562 cells after Stel B treatment. The Stel B-induced apoptosis might be in mitochondrial pathway, with increase of Bad and Bax, decrease of Bcl-2 and activation of caspase-9. In addition, dose-dependent increase of reactive oxygen species (ROS) and loss of mitochondrial membrane potential (MMP) occurred. Meanwhile, Stel B inhibited phosphorylation of Stat5, expression of 4 PI3K catalytic isoforms, and phosphorylation of the downstream effectors including PDK1 and Akt, suggesting that inhibition against Stat5 and PI3K might be involved in the apoptosis-inducing effect. Combination of Stel B with Imatinib with ratio as IC_50 Stel B_: 5×IC_50 Imatinib_ led to synergistic effect. Stel B might become a promising candidate for CML therapy alone or together with Imatinib.

## INTRODUCTION

Chronic myeloid leukemia (CML) is a myeloproliferative disease characterized by Philadelphia (Ph) chromosome. The oncoprotein Bcr-Abl, generated from Ph chromosome, is constitutively activated in CML and therefore leads to uncontrolled cell proliferation [1, 2]. Bcr-Abl inhibitors like Imatinib, Nilotinib and Dasatinib, highly improved the clinical therapy of CML in the past years. However, drug resistance, early relapse and persistence of leukemic stem cells led to the limited outcome of Bcr-Abl inhibitor monotherapy [3]. Therefore, discovery of novel agent is still urgent for CML treatment.

Natural products often serve as drug leads for cancer therapy because of their mechanistic diversity and good availability [4, 5]. Marine sponges are known to be productive source of anticancer drug leads [5–7]. We previously isolated Smenospongine and Aaptamine as antitumor compounds from marine sponge *Dactylospongia elegans* and *Aaptos suberitoides*, respectively [8, 9]. Stellettin B (Stel B) is an isomalabaricane triterpenoid that we isolated from marine sponge *Jaspis stellifera* [10]. As we reported previously, Stel B showed antiproliferative, cell cycle G1-arrest, apoptosis-inducing, as well as autophagy-inducing activities on human solid tumor cells including non-small cell lung cancer (NSCLC) A549 [11]. However, the antileukemia effect of Stel B on CML has not been reported to date.

Recently, we investigated the antileukemia activity of Stel B on human CML cells and the underlying mechanism. Combinational effect of Stel B and Imatinib on CML was also examined. Finally, we studied the activity of Stel B on multidrug resistant K562/A02 cells.

## RESULTS

### Growth inhibitory effect of Stel B on CML K562 and KU812 cells, lymphocyte U937 cells and normal PBMC (peripheral blood mononuclear cell) cells

**Figure 1:**
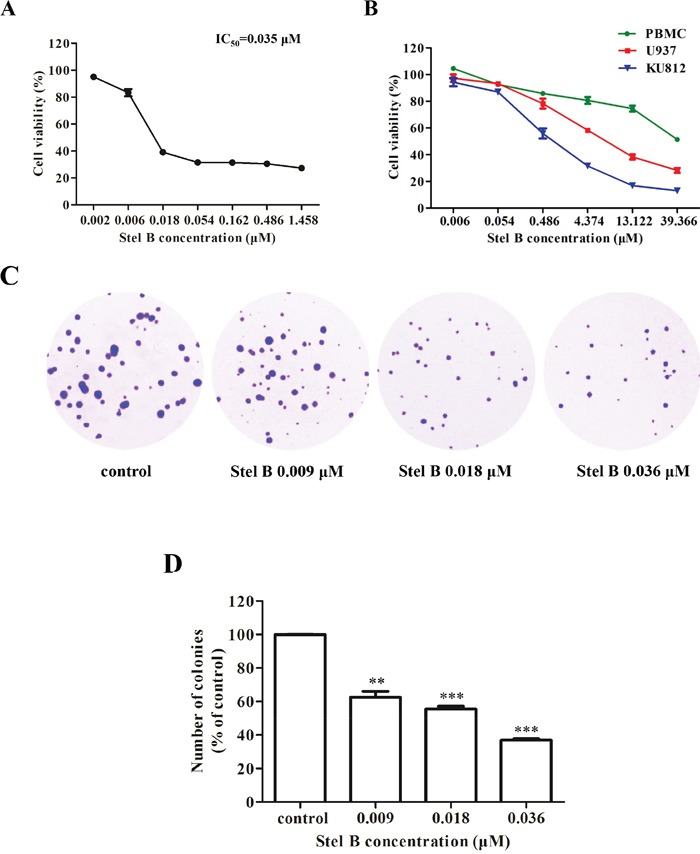
Potent Effect of Stel B on growth of CML cells **(A)** WST-8 assay. K562 cells were cultured in 96-well plates with 0, 0.002, 0.006, 0.018, 0.054, 0.162, 0.486, 1.458 μM of Stel B for 48 h, and cell viability was measured 4 h after addition of WST-8 reagent. **(B)** WST-8 assay. KU812, U937 and PBMC cells were cultured in 96-well plates with 0, 0.006, 0.054, 0.486, 4.374, 13.122, 39.366 μM of Stel B for 48 h, and cell viability was measured as described in Figure 1A. **(C)** Soft agar assay. After treatment with 0, 0.009, 0.018 and 0.036 μM of Stel B for 48 h, K562 cells were further grown in soft agar for 10 days, followed by staining with crystal violet. Colonies were counted under a microscope to determine the effect of Stel B on tumorigenicity of K562 cells. **(D)** Quantification of the colonies formed by K562 cells with or without Stel B treatment in soft agar. Data are expressed as mean ± SD, representative of three independent experiments. ^*^: *p* < 0.01, ^**^*: *p* < 0.001, compared with control.

K562 cells were exposed to various concentrations (0, 0.002, 0.006, 0.018, 0.054, 0.162, 0.486, 1.458 μM) of Stel B for 48 h, cell viability was determined by WST-8 assay. As shown in Figure 1A, Stel B reduced K562 cell viability in a dose-dependent manner. The IC_50_ value (half-maximal inhibitory concentration) was calculated to be 0.035 μM.

The effects of Stel B on the growth of another Ph-positive CML cell KU812, other type of leukemia cell line-histiocytic lymphocyte cell U937, as well as normal PBMC cells, were also investigated. As shown in Figure 1B, Stel B showed more potent growth inhibition against KU812 with an IC50 of 0.95 μM, than that against U937 with an IC50 of 4.55 μM. More interestingly, even after treatment with 39 μM of Stel B, less than 50% inhibition was observed for normal cell PBMC, suggesting low cytotoxicity of Stel B on normal cells.

Since K562 cell exhibited much higher response than KU812, we further investigated the antitumor effect of Stel B on CML by use of K562 cells. Firstly, soft agar colony formation assay (soft agar assay), which is known to be a reliable method to evaluate tumorigenicity of cancer cells [12], was used. After exposure to Stel B at 0, 0.009, 0.018 and 0.036 μM for 48 h, K562 cells were grown in soft agar for 10 days. As shown in Figure 1C and 1D, both number and size of the cell colonies were decreased remarkably by Stel B treatment in a dose-dependent manner, suggesting Stel B inhibited tumorigenicity of K562 cells.

### Stel B does not affect cell cycle distribution of K562 cells

**Figure 2:**
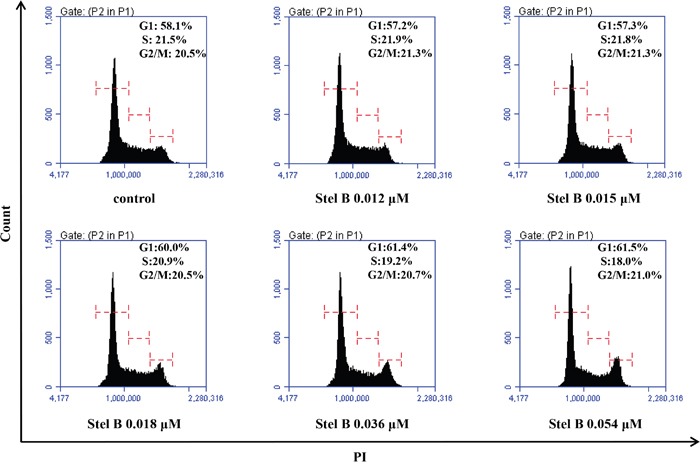
Cell cycle distribution of K562 cells with or without Stel B treatment Cells were exposed to different concentrations (0, 0.012, 0.015, 0.018, 0.036, 0.054 μM) of Stel B for 48 h. After PI staining, flow cytometry analysis was performed to determine cell cycle distribution. PI: propidium iodide.

Disturbance of cell cycle could inhibit cell growth. To assess the effect of Stel B on K562 cell cycle, we analyzed the cell cycle distribution by flow cytometric assay after PI staining of the cells with or without Stel B treatment. As shown in Figure 2, the cell population in G0/G1, S and G2/M phases is 61.5%, 18.0% and 21.0% respectively in 0.054 μM Stel B -treated cells, while that of untreated cells is 58.1%, 21.5% and 20.5%, suggesting no obvious change in cell cycle distribution was caused by Stel B treatment.

### Stel B induces apoptosis in K562 cells

**Figure 3:**
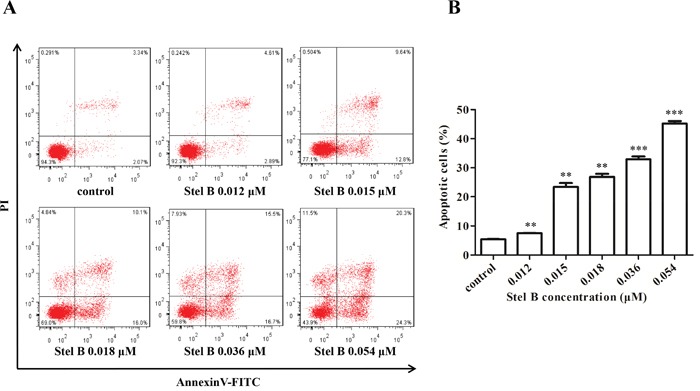
Apoptosis of K562 cells induced by Stel B **(A)** Flow cytometric analysis of cell apoptosis with Annexin V-FITC/PI double staining. K562 cells were harvested 48 h after treatment by indicated concentrations of Stel B, stained with Annexin V-FITC/PI and subjected to flow cytometry analysis. **(B)** Quantification of the apoptotic cells in both early and late stage. Data represent mean± SD of three independent experiments. ^*^: *p* < 0.01, ^**^*: *p* < 0.001, compared with control.

To investigate whether the reduction of cell viability in Stel B-treated K562 cells was caused by apoptosis induction, the apoptotic cell population was determined by Annexin V-FITC/PI double staining. As indicated in Figure 3A and 3B, Stel B triggered apoptosis at both early (lower-right quadrant) and late (upper-right quadrant) stage in K562 cells in a dose-dependent manner.

**Figure 4:**
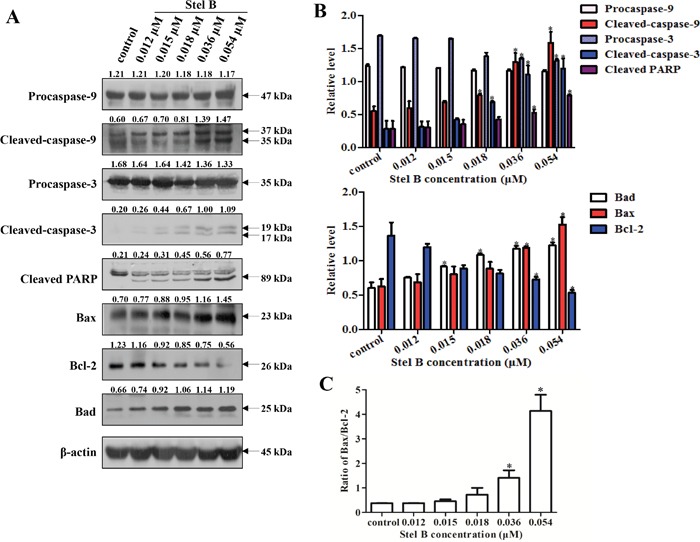
Effect of Stel B on the expression of apoptosis-related proteins in K562 cells **(A)** Expression of the apoptosis-related proteins detected by Western blot in K562 cells after Stel B treatment. K562 cells were treated with indicated concentrations of Stel B for 48 h. The cells were collected to be available for Western blot detection of caspase-9, caspase-3, and PARP, Bcl-2, Bax and Bad. β-actin was used as the loading control. **(B)** Bar graphs show the relative levels of Procaspase-9, Cleaved-caspase-9, Procaspase-3, Cleaved-caspase-3, and Cleaved PARP (upper panel), as well as Bcl-2, Bax and Bad (lower panel). Data are mean ± SD, representative of three independent experiments. *: *p* < 0.05, compared with control. **(C)** Determination of the Bax/Bcl-2 ratio as a well-known apoptosis marker. Data are mean ± SD, representative of three independent experiments. *: p < 0.05, compared with control.

To investigate the possible mechanism for Stel B to induce apoptosis, the expression of apoptosis regulators was analyzed by Western blot. As shown in Figure 4A and 4B, caspase-9, caspase-3 and PARP were activated in response to Stel B treatment, as the cleaved forms were increased. Reduction of Bcl-2 and enhancement of Bax as well as Bad were observed, together with an increase of Bax/Bcl-2 ratio (Figure 4C). These results suggest that the apoptosis induced by Stel B might be intrinsic in a mitochondria pathway.

**Figure 5:**
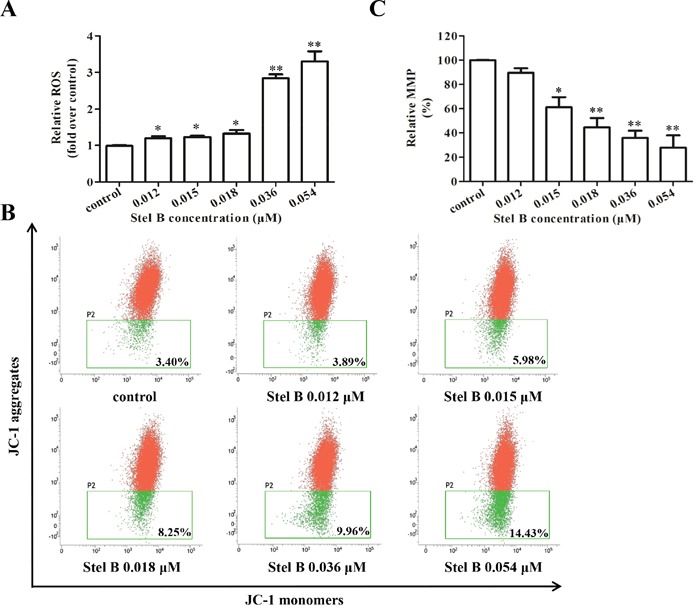
Effect of Stel B on the ROS generation and MMP in K562 cells **(A)** Flow cytometric determination of ROS generation in K562 cells with or without Stel B treatment. Cells were treated with indicated concentrations of Stel B for 48 h, followed by DCFH-DA staining for 30 min. The ROS levels were determined by flow cytometer. Data represent mean ± SD of three independent experiments. *: *p* < 0.05, ^*^: *p* < 0.01, compared with control. **(B)** Effect of Stel B on MMP in K562 cells with or without Stel B treatment. Cells were exposed to various concentrations of Stel B for 24 h. MMP (ΔΨ_m_) was measured by the uptake of JC-1 using flow cytometer. Cells containing JC-1 monomers (green) indicate low ΔΨ_m_ while those containing JC-1 aggregates (red) indicate high ΔΨ_m_. **(C)** Quantification of the MMP (% control) in K562 cells with or without Stel B treatment. MMP was calculated as the ratio of the red fluorescence to green fluorescence. Relative MMP represents the MMP of the respective sample over that of control (%). Data represent mean ± SD of three independent experiments. *: *p* < 0.05, ^*^: *p* < 0.01, compared with control.

It is known that reactive oxygen species (ROS) generation can cause mitochondrial dysfunction and DNA damage, thereby leading to programmed cell death including apoptosis [10, 13]. To examine whether the apoptosis induced by Stel B was related to ROS generation, the intracellular ROS was measured by flow cytometer with fluorescent reagent DCFH-DA. As shown in Figure 5A, after treatment with 0.012, 0.015, 0.018, 0.036, 0.054 μM of Stel B for 48 h, the amount of ROS increased dose dependently, suggesting that ROS production might be related to the apoptosis induced by Stel B. Mitochondrial dysfunction characterized by loss of mitochondrial membrane potential (MMP, Δψ_m_) is known to play a crucial role in the intrinsic apoptosis [14]. After treatment with 0.012, 0.015, 0.018, 0.036 and 0.054 μM of Stel B for 24 h, population of cells containing JC-1 monomers increased while the number of cells with JC-1 aggregates decreased (Figure 5B). The relative MMP of cells treated by Stel B reduced dose dependently (Figure 5C).

### Stel B inhibits PI3K/Akt pathway and the phosphorylation of transcription factor Stat5

**Figure 6:**
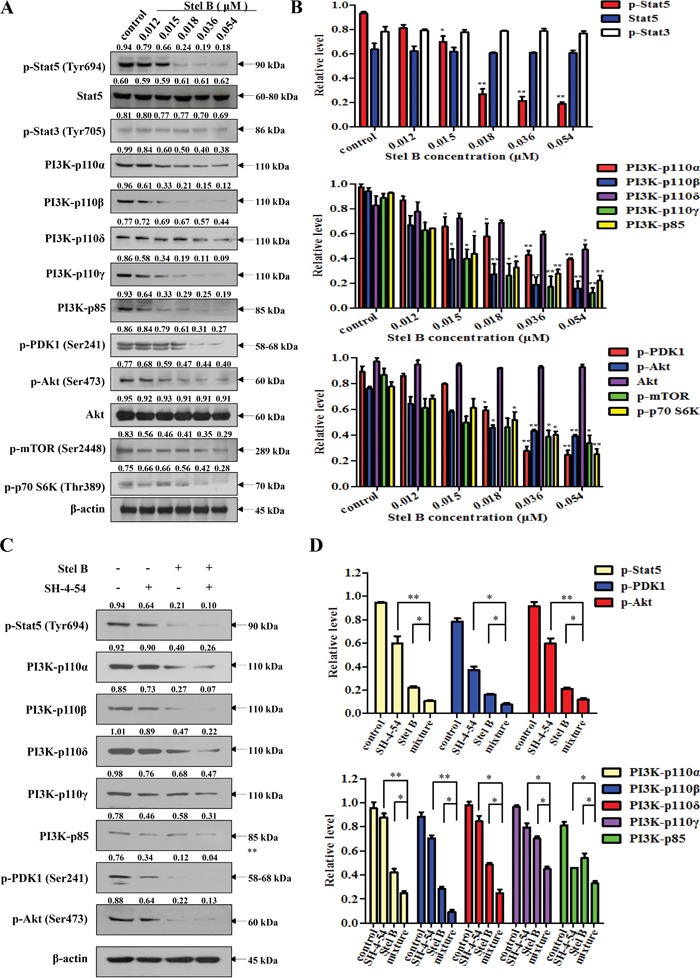
Effect of Stel B on Stat5, PI3K and the downstream effectors in PI3K/Akt pathway in K562 cells **(A)** K562 cells were treated with indicated concentrations of Stel B for 48 h. The treated cells were subjected for Western blot analysis of p-Stat5, Stat5, p-Stat3, PI3K-p110α, PI3K-p110β, PI3K-p110δ, PI3K-p110γ, PI3K-p85, p-PDK1, p-Akt, Akt, p-mTOR and p-p70 S6K. β-actin was used as the loading control. **(B)** Bar graphs show the relative levels of p-Stat5, Stat5 and p-Stat3 (upper panel), PI3K-p110α, PI3K-p110β, PI3K-p110δ, PI3K-p110γ and PI3K-p85 (middle panel), as well as p-PDK1, p-Akt, Akt, p-mTOR and p-p70 S6K (lower panel). Data are mean ± SD, representative of three independent experiments. *: *p* < 0.05, ^*^: *p* < 0.01, compared with control (without treatment). **(C)** K562 cells were treated with Stel B (0, 0.036 μM) in the presence or absence of SH-4-54 (3.2 μM) for 48 h. Signals of p-Stat5, PI3K-p110α, PI3K-p110β, PI3K-p110δ, PI3K-p110γ, PI3K-p85, p-PDK1 and p-Akt were determined by Western blot. β-actin was used as the loading control. **(D)** Bar graphs show the relative levels of p-Stat5, p-PDK1 and p-Akt (upper panel), PI3K-p110α, PI3K-p110β, PI3K-p110δ, PI3K-p110γ and PI3K-p85 (lower panel). Data are mean ± SD, representative of three independent experiments. *: *p* < 0.05, ^*^: *p* < 0.01, compared with mixture.

We previously reported that Stel B inhibited PI3K/Akt pathway in human NSCLC A549 cells [11]. To examine whether Stel B also inhibits PI3K/Akt pathway in K562 cells, phosphorylation of the downstream effectors in the pathway was first investigated by Western blot. As shown in Figure 6A and 6B, dose-dependent reduction of p-PDK1, p-Akt, p-mTOR and p-p70 S6K was observed after Stel B treatment for 48 h. We then determined the effect on the expression of the upstream PI3K including the catalytic subunit PI3K-p110α, PI3K-p110β, PI3K-p110δ, PI3K-p110γ, as well as the regulatory subunit PI3K-p85. Interestingly, the expressions of all the PI3K subunits were inhibited by Stel B dose-dependently (Figure 6A and 6B).

Since Stat5 was reported to regulate the expression of PI3K-p110 as well as PI3K-p85 [15], we then investigated the effect of Stel B on the phosphorylation of Stat5 in K562 cells. Figure 6A and 6B showed that p-Stat5 was significantly downregulated while p-Stat3 was not affected by Stel B treatment, suggesting that inhibition against Stat5 phosphorylation might contribute to the action of Stel B on PI3K expression and the phosphorylation of the downstream effectors like Akt. Similar effect on Stat5 was confirmed in KU812 cells (Supplementary Figure 1). Treatment with Stat5 inhibitor SH-4-54 alone and together with Stel B further supported the close relation of Stat5 and PI3K. As shown in Figure 6C and 6D, phosphorylation of Stat5 was reduced by SH-4-54, accompanied with downregulation of PI3K/Akt downstream effectors like PI3K-p110α, PI3K-p110β, PI3K-p110δ, PI3K-p110γ, PI3K-p85, p-PDK1 and p-Akt. Co-treatment with Stel B enhanced the above effect, supporting that inhibition of PI3K/Akt pathway might be at least partially attributed to the action on Stat5. To further verify this hypothesis, we knocked down Stat5 to see the effect on PI3K/Akt pathway. As shown in Supplementary Figure 2A and 2B, silencing of Stat5 significantly reduced PI3K expression including both catalytic subunit PI3K-p110β and regulatory subunit PI3K-p85. In addition, Stat5 siRNA enhanced the inhibitory action of Stel B on PI3K expression, further supporting that the inhibition against Stat5 might contribute to the effect on PI3K/Akt pathway.

### Synergistic effect of Stel B and Imatinib on K562 cells

**Figure 7:**
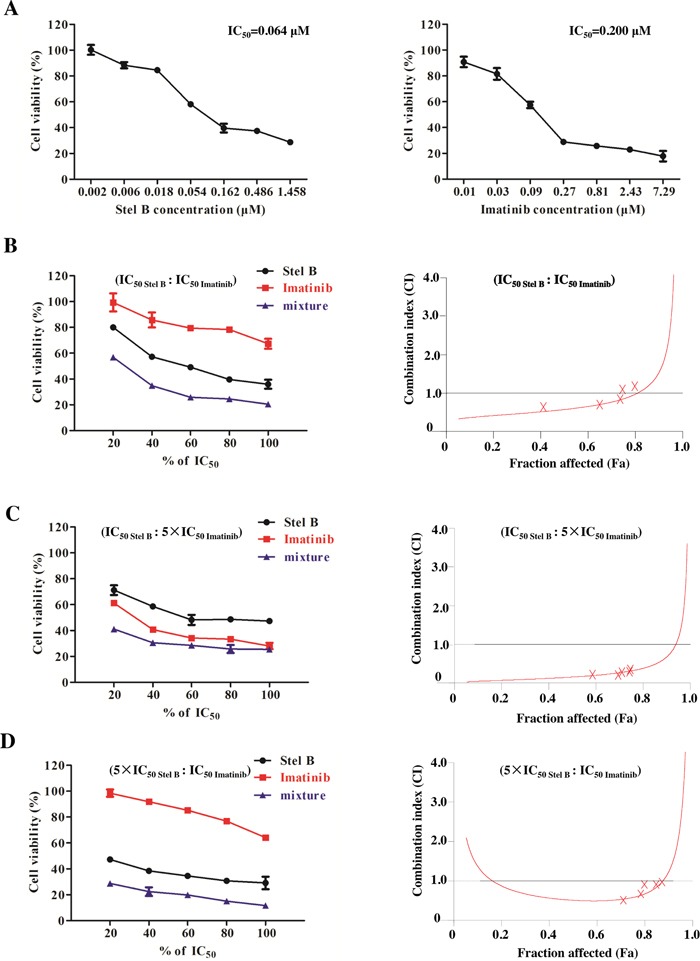
Synergistic effect of Stel B and Imatinib in K562 cells **(A)** Growth inhibitory activity of Stel B and Imatinib on K562 cells determined by MTT assay. K562 cells were exposed with indicated concentrations of Stel B or Imatinib. Cell viability was determined by MTT assay. Data are mean ± SD, representative of three independent experiments. **(B, C, D)** K562 cells were incubated with various concentrations of Stel B and/or Imatinib (20%, 40%, 60%, 80%, 100% IC_50_ of each drug) for 48 h, Three fixed ratios of IC_50 Stel B_: IC_50 Imatinib_, IC_50 Stel B_: 5×IC_50 Imatinib_, 5×IC_50 Stel B_: IC_50 Imatinib_ were used. Cell viability after different treatments was determined by MTT assay (left). Data are mean ± SD, representative of three independent experiments. Combinational effect was analyzed using CalcuSyn software and the resulting CI-Fa plots are shown (right). The horizontal line of CI = 1, representing additivity, is indicated. Values of drug combinations below the horizontal line indicate synergism. CI: combination index, Fa: Fraction affected.

Currently Imatinib is still the first line drug in clinic for CML therapy. To examine whether combination of Stel B and Imatinib could show better therapeutic efficacy, we performed synergisitc assay by using Chou and Talalay's method [16]. We first determined antiproliferative activities of Stel B and Imatinib with MTT assay, and calculated the IC_50_ values to be 0.064 μM and 0.200 μM, respectively (Figure 7A). Then we analyzed the combinatory effect by using a series of drug combinations (20%, 40%, 60%, 80%, 100% of each drug). Three constant ratios of IC_50 Stel B_: IC_50 Imatinib_, IC_50 Stel B_: 5×IC_50 Imatinib_, 5×IC_50 Stel B_: IC_50 Imatinib_ were used. As shown in Figure 7B, 7C and 7D (left panel), all of the 3 drug combinations indicated greater growth inhibitory effect than either drug alone. Then combination index (CI) profiles were prepared, and the values at ED_50_, ED_75_ and ED_90_ were calculated with CalcuSyn software. Combination of IC_50 Stel B_: 5×IC_50 Imatinib_ produced synergism at ED_50_, ED_75_ and ED_90_ (CI = 0.154, 0.313 and 0.670, respectively), while drug combinations at other 2 ratios exhibited antagonistic effect at ED_90_ (Figure 7B, 7C and 7D, Table 1). Even at ED_50_, combination of IC_50 Stel B_: IC_50 Imatinib_ and 5×IC_50 Stel B_: IC_50 Imatinib_ showed weaker synergism (CI = 0.647 and 0.513, respectively) (Figure 7C and 7D, Table 1). Therefore, IC_50 Stel B_: 5×IC_50 Imatinib_ combination exhibits the best synergistic effect.

**Table 1:** Combination indexes (CI) for Stel B and Imatinib

Drug or drug combination	Parameters		CI values (mean±SD)		
	D_m_ (mean±SD)	r	ED_50_	ED_75_	ED_90_
Stel B	0.064±0.052	0.978	-	-	-
Imatinib	0.200±0.024	0.976	-	-	-
Stel B+ Imatinib (IC_50_: IC_50_)	0.042±0.015	0.982	0.647±0.112	0.991±0.045	1.714±0.083
Stel B+ Imatinib (IC_50_: 5×IC_50_)	0.014±0.012	0.975	0.154±0.011	0.313±0.006	0.670±0.041
Stel B+ Imatinib (5×IC_50_: IC_50_)	0.041±0.052	0.975	0.513±0.045	0.604±0.072	1.438±0.171

**Figure 8:**
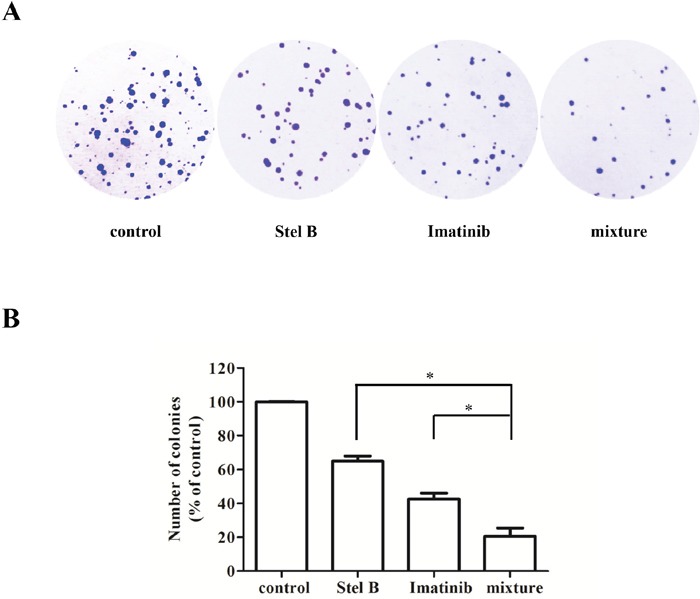
Effect of the combination of Stel B and Imatinib on tumorigenicity of K562 cells **(A)** The cells were treated with Stel B (0.0064 μM) and/or Imatinib (0.1 μM) for 48 h, and grown in soft agar at 37°C. Ten days later, colonies more than 0.1 mm in diameter were counted using Image J software. **(B)** Quantification of the colonies formed by K562 cells in soft agar after treatment with Stel B and Imatinib alone or in combination. Data are mean ± SD, representative of three independent experiments. *: p < 0.05, compared with mixture (combination).

**Figure 9:**
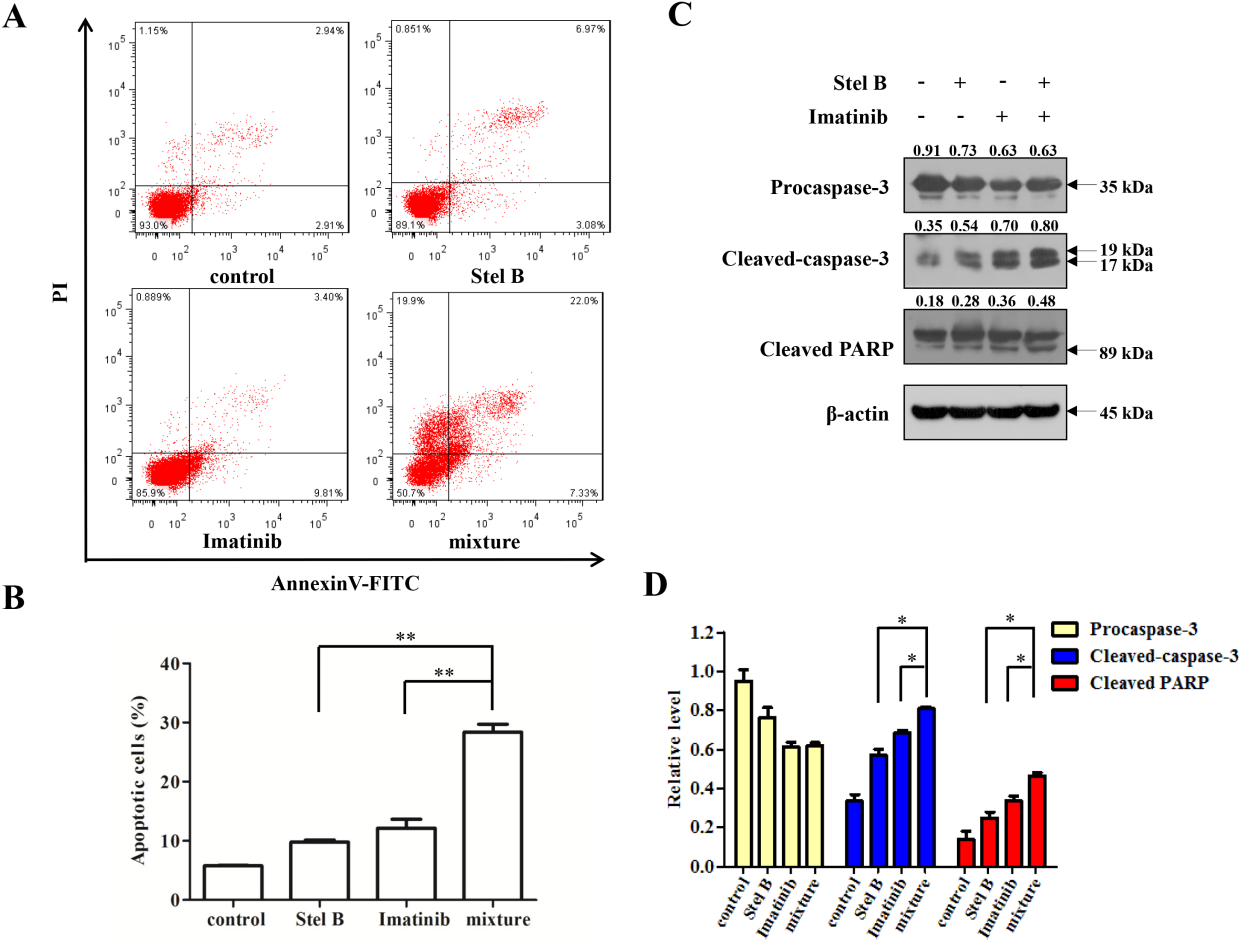
Effect of the combination of Stel B and Imatinib on apoptosis in K562 cells **(A, B)** K562 cells exposed with Stel B (0.0064 μM) and/or Imatinib (0.1 μM) for 48 h, were stained with Annexin V-FITC/PI and analyzed using flow cytometer. Data represent mean ± SD of three independent experiments. ^*^: p < 0.01, compared with mixture (combination). **(C)** After treatment with Stel B (0.0064 μM) and/or Imatinib (0.1μM) for 48 h, cleavage of caspase-3 and PARP of K562 cells was analyzed by Western blot. β-actin was used as the loading control. **(D)** Bar graphs show the relative levels of Procaspase-3, Cleaved-caspase-3 and Cleaved PARP. Data are mean ± SD, representative of three independent experiments. *: *p* < 0.05, compared with mixture.

To confirm the antiproliferative effect of IC_50 Stel B_: 5×IC_50 Imatinib_ combination, soft agar assay was carried out. Combination of Stel B (0.0064 μM) and Imatinib (0.1 μM) significantly decreased the number of colonies, compared with either drug alone (Figure 8A and 8B). Moreover, apoptosis-inducing activity was also examined with flow cytometer after Annexin V-FITC/PI staining. As indicated in Figure 9A and 9B, co-treatment with 0.0064 μM of Stel B and 0.1 μM of Imatinib led to a highly enhanced apoptosis-inducing effect, compared with either single treatment. In addition, increased cleavage of caspase-3 and PARP was also observed for the combination (Figure 9C and 9D).

### Stel B indicates lower activity on multidrug resistant K562/A02 cells

**Figure 10:**
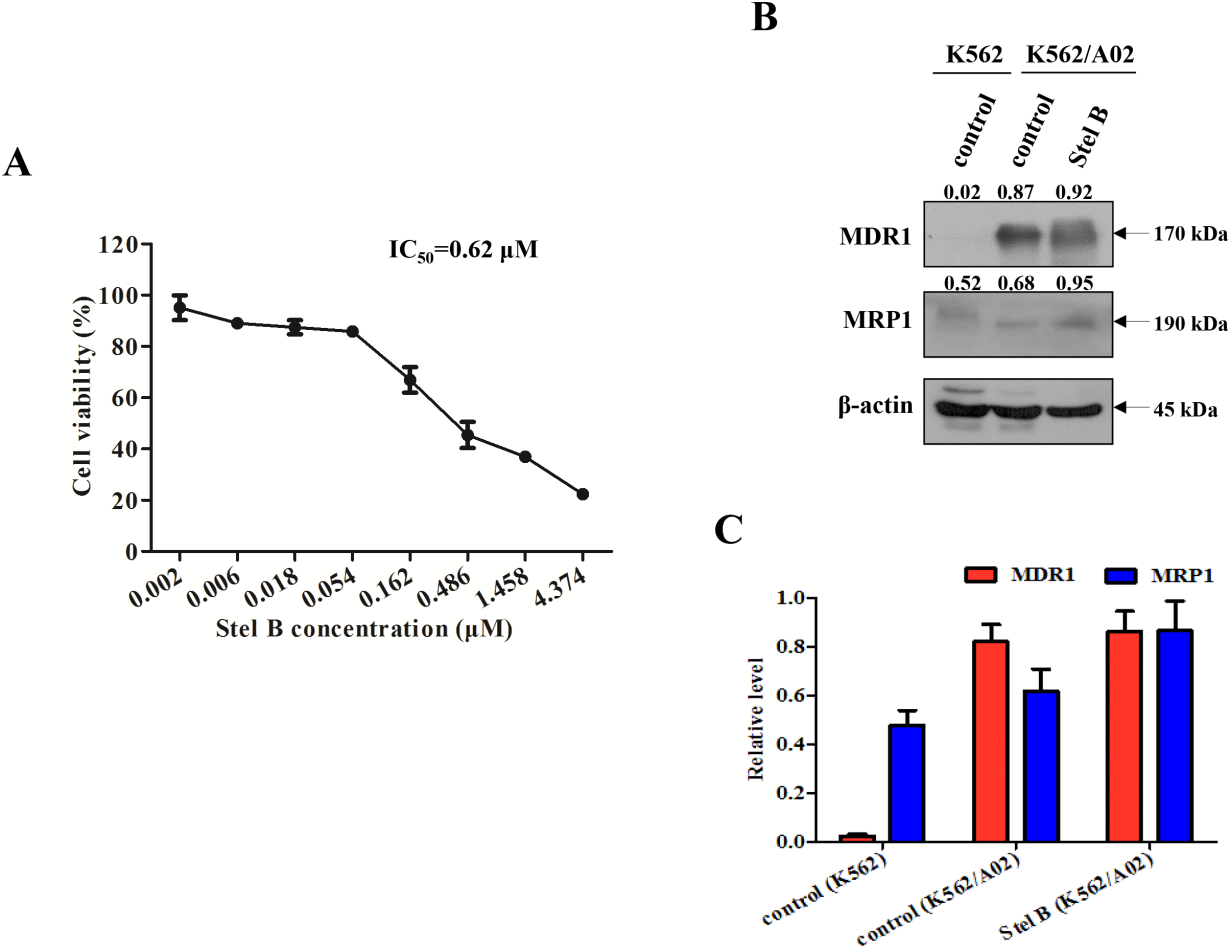
Effect of Stel B on the growth of multidrug resistant K562/A02 cells **(A)** K562/A02 cells in 96-well plates were treated with 0, 0.002, 0.006, 0.018, 0.054, 0.162, 0.486, 1.458, 4.374 μM of Stel B for 48 h. Cell viability was measured by MTT assay. **(B)** Expression level of multidrug resistance related proteins in K562 and K562/A02 cells. Expression of MDR1 and MRP1 in K562 cells, and K562/A02 cells with or without Stel B (0.62 μM) treatment was analyzed by Western blot. β-actin was used as the loading control. **(C)** Bar graphs show the relative levels of MDR1 and MRP1. Data are mean ± SD, representative of three independent experiments.

To examine whether Stel B exhibits equal activity on multidrug resistant tumor cells to that on the parent one, we determined the antiproliferative effect on K562/A02, which is an ADR-selected MDR cell sub-line. As shown in Figure 10A, while Stel B showed inhibition against K562/A02 proliferation, the IC_50_ was calculated to be 0.62 μM, about 10 folds over that for the parent K562 cells, suggesting that the multidrug resistant cells might be less sensitive to Stel B. To support this prediction, Western blot was carried out to detect multidrug resistant proteins MDR1 and MRP1. Figure 10B and 10C indicated that expression of both MDR1 and MRP1 was higher in K562/A02 cells than in K562 cells, and 0.62 μM of Stel B treatment did not reduce the expression of MDR1 nor MRP1, suggesting MDR1 or MRP1 might be the cause of resistance for K562/A02 cells to Stel B.

## DISCUSSION

In this current study, we demonstrated that Stel B inhibited the proliferation of K562 CML cells potently with an IC_50_ of low nM. After treatment for 48 h, Stel B induced obvious apoptosis in both early and late stage while no obvious effect on cell cycle was observed in K562 cells. We previously reported that Stel B induced G1 cell cycle arrest, together with the apoptosis in late stage of NSCLC A549 cells [11]. Such different effects might be attributed to different genetic characteristics of the two cell lines.

Apoptosis is known to be mediated by the mitochondrial pathway and the death receptor pathway. Bcl-2 family members are crucial mediators of apoptosis in mitochondrial pathway [17, 18]. Based on Bcl-2 homology (BH) region, structure and function, this family is divided into pro-apoptotic proteins (Bax, Bak), anti-apoptotic proteins (Bcl-2, Bcl-xL) and BH3-only proteins (Bid, Bad, Bim) [18]. By forming homo- or hetero-oligomers, Bax and Bak insert and permeabilize mitochondrial outer membrane [18], leading to release of cytochrome C and activation of caspase-9 [19]. The activated caspase-9 in turn promotes the cleavage of caspase-3 as well as its substrate PARP. On the other hand, Bcl-2 is known to inhibit Bax/Bak complex formation, while such activity can be neutralized by Bad [20, 21]. In our study, Bad and Bax were upregulated, while Bcl-2 was downregulated in K562 cells after Stel B treatment. Consistently, the cleaved caspase-9, caspase-3 and PARP were increased. Therefore, Stel B might induce mitochondrial apoptosis via activating Bad/Bax/Caspase-9/Caspase-3/PARP pathway. By oxidation of the mitochondrial proteins, ROS is known to induce mitochondrial outer membrane permeabilization (MOMP) and loss of MMP [14]. We then examined whether ROS generation is involved in Stel B-induced apoptosis or not. Our result indicated that Stel B treatment enhanced ROS generation and reduced MMP in a dose dependent manner, supporting that the Stel B-induced apoptosis in K562 cells might be mediated by ROS-involved mitochondrial pathway.

To investigate the antiproliferative and apoptosis-inducing mechanism of Stel B, we first examined the activity on PI3K/Akt pathway since we previously reported the inhibition of PI3K/Akt pathway by Stel B in A549 cells. As expected, phosphorylation of PDK1, Akt and the downstream effectors including mTOR and p70 S6K, was inhibited by Stel B treatment. We previously reported Stel B exhibited no inhibition against PI3K activity in kinase assay [10]. Then we examined whether Stel B inhibited the expression of PI3K including four p110 catalytic subunits and p85 regulatory subunit. Interestingly, Stel B treatment reduced the expression of all the four p110 catalytic subunits and the p85 regulatory subunit. It was reported that Stat5 could promote the expression of both p110 and p85, as a transcription factor [15]. We then examined the effect of Stel B on the phosphorylation of Stat5 together with Stat3. Our results showed that Stel B indeed inhibited phosphorylation of Stat5, while Stat3 was almost not affected. It has been known that only Stat5 was constitutively activated in CML cells [22, 23], as a downstream effector of Bcr-Abl [24]. Stel B might inhibit phosphorylation of Stat5 via suppressing the upstream molecules mediated by Bcr-Abl. Furthermore, Stat5 inhibitor SH-4-54 also reduced expression of PI3K catalytic subunits and regulatory subunits, as well as phosphorylation of the downstream molecules like Akt, and enhanced the effect of Stel B. Silencing of endogenous Stat5 by using siRNA significantly reduced PI3K expression, and enhanced the action of Stel B. These results suggest the effect of Stel B on PI3K/Akt pathway might be attributed to the inhibition against Stat5. Taken together, Stel B might inhibit the phosphorylation of Stat5, and therefore block the expression of PI3K, leading to inactivation of Akt pathway and apoptosis in K562 cells. However, the initial target of Stel B remains unclear yet, identification of which is to be done by use of chemical proteomic approach with labeled Stel B as probe.

While Imatinib has highly improved the clinical therapy of CML, resistance remains a barrier to better patient outcomes [3, 25]. Drug combination with lower doses might improve the therapeutic index while keeping low toxicity [26]. Our result showed that combination of Imatinib and Stel B at a ratio of IC_50 Stel B_: 5×IC_50 Imatinib_ exhibited synergistic effect, while the combinations with other two ratios did not always lead to synergism, suggesting that combination of Stel B and Imatinib might be a potential approach for CML therapy when approved in the future, while the dose ratio for the two drugs must be designed carefully.

Multidrug resistance is an obstacle for cancer chemotherapy, which usually occurs after a long term treatment. Such patients are not only insensitive to the administered drug but also to other antitumor agents, due to the overexpression of multidrug resistant proteins like MDR1 and MRP1. To investigate whether Stel B shows antitumor activity on multidrug resistant CML, we determined the antiproliferative effect on K562/A02 as well as on its parent cell line K562. While Stel B inhibited the proliferation of K562/A02, the activity (IC_50_: 0.62 μM) was obviously weaker than that for K562 cells (IC_50_: 0.064 μM), suggesting that higher doses are needed for Stel B monotherapy of the multidrug resistant CML patients when approved.

In conclusion, Stel B showed potent *in vitro* antitumor activity on CML K562 cells. Mitochondrial apoptosis was induced, which might be attributed to the inhibition of Stat5 phosphorylation and PI3K expression. Combination with Imatinib at ratio of IC_50 Stel B_: 5×IC_50 Imatinib_ led to synergism in the antiproliferative effect. Stel B has the potential to become a drug candidate for CML therapy, while further evaluation must be done.

## MATERIALS AND METHODS

### Reagents

Stel B was isolated by us from *Jaspis stellifera* and structurally identified as described previously [10]. Imatinib and SH-4-54 was purchased from Selleck (London, ON, Canada). WST-8 assay kit was from Dojindo Laboratories (Kumamoto, Japan). FITC Annexin V Apoptosis Detection Kit was obtained from BD Biosciences (San Jose, CA, USA). Propidium iodide (PI) and 2′,7′-Dichlorofluorescein diacetate (DCFH-DA) were purchased from Sigma-Aldrich (St. Louis, MO, USA). Lipofectamine 2000 was from Life Technologies (Carlsbad, CA, USA). 5,5′,6,6′-tetrachloro-1, 1′,3,3′-tetraethylbenzimidazole-carbocyanide iodine (JC-1) was purchased from Molecular Probes (Eugene, OR, USA). MTT (3-(4,5-dimethyl-2-thiazolyl)-2,5-diphenyl-2-H-tetrazolium bromide) was from Amresco (Solon, OH, USA). Antibodies against phospho-Stat5 (Tyr694), phospho-Stat3 (Tyr705), PI3K-p110α, PI3K-p110β, PI3K-p110δ, PI3K-p110γ, PI3K-p85, phospho-PDK1 (Ser241), phospho-Akt (Ser473), Akt, phospho-mTOR (Ser2448), phospho-p70S6 Kinase (Thr389), caspase-3, caspase-9, poly (ADP-ribose) polymerase (PARP), β-actin, anti-mouse and anti-rabbit HRP-conjugated secondary antibodies were obtained from Cell Signaling Technology (Danvers, MA, USA). Antibodies against MDR1, MRP1, Bad, Bcl-2 and Bax were from Santa Cruz Biotechnology (Santa Cruz, CA, USA).

### Cell culture

Human chronic myeloid leukemia K562 and KU812, as well as human histiocytic lymphocyte cell U937, were purchased from Cell Resource Center, Peking Union Medical College (Beijing, China). Cells were cultured in RPMI medium containing 10% fetal bovine serum, 1% kanamycin (100 μg/ml) and 1% glutamine (0.44 μg/ml) at 37°C in a humidified atmosphere containing 5% CO_2_. Peripheral blood mononuclear cells (PBMCs) were obtained from total blood samples which were collected from healthy volunteers who have signed Institutional Review Board Approval and Informed Consent Forms (Tianjin Medical University, China). The PBMCs were isolated by using Human Lymphocyte Separation Medium (Dakewe, Beijing, China) and cultured in RPMI medium containing 20% fetal bovine serum, 1% kanamycin (100 μg/ml), and 1% glutamine (0.44 μg/ml) at 37°C in a humidified atmosphere containing 5% CO_2_. K562/A02, an ADR-selected multidrug resistance (MDR) cell sub-line, was kindly provided by Institute of Hematology, Chinese Academy of Medical Sciences. To maintain the MDR phenotype, K562/A02 cells were routinely cultured with 1 μg/ml of Adriamycin (ADR). Two weeks before experiment, the medium was changed to that of ADR-free.

### Cell viability assay

Cell viability was determined using WST-8 and MTT assay, respectively, as described [27, 28]. For WST-8 assay, two hundred μl of the suspension of the respective cells was seeded into 96-well plate and exposed to various concentrations of Stel B. Forty eight hours later, 10 μl of WST-8 was added to each well. After further incubation for 4 h, the absorbance at 450 nm was measured using microplate reader iMark (Bio Rad, Hercules, CA, USA). In the case of MTT assay, K562 and K562/A02 cells were seeded at a density of 4 × 10^3^ cells per well (200 μl) and treated with Stel B or Imatinib for 48 h. Then, 20 μl of MTT (5 mg/ml) was added. Four hours later, the absorbance at 490 nm was measured with microplate reader iMark.

### Soft agar assay

Soft agar assay was used to determine tumorigenicity of K562 cells as reported [29] with a small modification. After pre-treatment with Stel B or Imatinib for 48 h, the assays were carried out in 60-mm dishes in which there was 4 ml of 0.6% agarose in the bottom with 2 ml of 0.3% agarose containing the cells (1.2 × 10^4^ cells/dish) on the top. After culture for 10 days at 37°C with 5% CO_2_, the colonies were fixed with 4% paraformaldehyde followed by 30 min of crystal violet (0.5%) staining. Colonies larger than 0.1 mm in diameter were counted using Image J software (NIH, Bethesda, MD, USA). At least three parallel dishes were scored for each treatment.

### Cell cycle distribution analysis

The effect of Stel B on cell cycle distribution was analyzed by flow cytometer as we previously described [9, 30]. K562 cells were treated with different concentrations of Stel B (0, 0.012, 0.015, 0.018, 0.036, 0.054 μM) for 48 h, fixed with 70% ethanol, washed twice with PBS and stained with PI solution (25 μg/ml). After incubation for 30 minutes in the dark at 4°C, the cells were analyzed by flow cytometer BD Accuri C6 (BD Biosciences, San Jose, CA, USA).

### Flow cytometric analysis of apoptosis with Annexin V-FITC/PI staining

Analysis of apoptosis was carried out by Annexin V-FITC/PI double staining as described by us previously [31]. Briefly, K562 cells (4 × 10^5^ cells/ml, 2 ml) were plated in 6-well plates and treated with Stel B, Imatinib or their combination for 48 h. After harvested, the cells were resuspended in 100 μl of binding buffer, incubated with Annexin V-FITC/PI solution in the dark for 15 min. Finally, samples were analyzed by flow cytometer FACS Verse (BD Biosciences, San Jose, CA, USA). Data were quantified by using Flow Jo Software (Tristar, CA, USA).

### Flow cytometric analysis of ROS production

Intracellular ROS level was measured using DCFH-DA fluorescent probe as we reported [31]. After exposure to Stel B (0, 0.012, 0.015, 0.018, 0.036, 0.054 μM) for 48 h, K562 cells were incubated with 10 μM of DCFH-DA for 30 min at 37°C. The resulting fluorescent intensity (λ_ex_: 488 nm, λ_em_: 530 nm) was measured using flow cytometer FACS Verse.

### Mitochondrial membrane potential (MMP, Δψ_m_) assay

JC-1, which is a cell-penetrating lipophilic cationic fluorochrome, was used to determine the MMP. The K562 cells (4 × 10^5^ cells/ml) were plated in 6-well plate, treated with Stel B (0, 0.012, 0.015, 0.018, 0.036, 0.054 μM) for 24 h. After washing with ice-cold PBS, cells were stained with JC-1 (2 μM) for 20 min at 37°C. The resulting fluorescence at 530 nm (green) and 590 nm (red) was measured using flow cytometer FACS Verse. Cells containing JC-1 aggregates have high Δψ_m_ and show red fluorescence. Cells with low Δψ_m_ are those containing JC-1 monomeric form, and show green fluorescence. The ratio of red fluorescence to green fluorescence was calculated as MMP (Δψ_m_). Relative MMP represents the MMP of the respective sample over that of control (%).

### Western blot analysis

Western blot analysis was performed as we described previously [32, 33]. After 48 h of drug treatment, the cells were collected and lysed using RIPA lysis buffer (Roche Diagnostics, Basel, Switzerland). Protein concentrations were determined with BCA Protein Assay Kit (Pierce, Rockford, IL, USA). Equal amounts of protein were subjected to 10% sodium dodecyl sulfate polyacrylamide gel electrophoresis (SDS-PAGE) and transferred to PVDF membranes (Millipore, Billerica, MA, USA). After treatment with 5% non-fat milk for 1 h, the blots were incubated with specific primary antibodies at 4°C overnight, then exposed to the respective HRP - conjugated secondary antibodies for 1 h. The resulting protein bands were visualized with ECL system and analyzed using Image J software.

### Stat5 siRNA transfection

Human Stat5 siRNA or scrambled siRNA (Invitrogen, Carlsbad, CA, USA) transfection was performed using Lipofectamine 2000 reagent according to the manufacturer's instructions. K562 cells (6 × 10^5^ cells/ml) were seeded in 6-well plate with 1 ml of RPMI medium without antibiotics. Twenty nanomoles of Stat5A siRNA (sense 5′-GCAACCUGUGGAACCUGAAACCAUU-3′ and antisense 5′-AAUGGUUUCAGGUUCCACAGGU UGC-3′) and Stat5B siRNA (sense 5′-UCCCUGCG AGUCUGCUACUGCUAAA-3′ and antisense 5′-UUUAGCAGUAGCAGACUCGCAGGGA-3′), or scrambled siRNA diluted in Opti-MEM medium was mixed gently with Lipofectamine 2000 and incubated for 20 min at room temperature. Then, transfection was performed by adding the mixture drop-wise to the cells, followed by incubation for 6 h. After replacement of the transfection mixture with fresh RPMI medium containing antibiotics and FBS, the transfected cells were used for Western blot analysis.

### Synergism assay

Drug synergy was evaluated as reported by us previously [30], based on Chou and Talalay method [16]. Briefly, K562 cells were treated for 48 h with Stel B and Imatinib at three constant ratios (IC_50 Stel B_: IC_50 Imatinib_, IC_50 Stel B_: 5×IC_50 Imatinib_, 5×IC_50 Stel B_: IC_50 Imatinib_). Data obtained from the growth inhibitory experiments were analyzed by CalcuSyn software to determine drug combination effect. Combination indexes (CI) were then calculated, with CI < 1, CI = 1 and CI > 1 indicating synergism, additivity and antagonism, respectively. All experiments were carried out in triplicate.

### Statistical analysis

Data are presented as mean ± standard deviation (SD), representative of at least three independent experiments. Student's t-test was carried out by use of GraphPad Prism 5 software (GraphPad, San Diego, CA, USA). Value of p< 0.05 was considered statistically significant.

## SUPPLEMENTARY MATERIALS FIGURES AND TABLES


