# Supplementary material for: Stellettin B induces apoptosis in human chronic myeloid leukemia cells via targeting PI3K and Stat5
Source: Oncotarget. 2017 Mar 7;8(17):28906–21. doi: 10.18632/oncotarget.15957 (PMC5438702; doi:10.18632/oncotarget.15957)
Supplement: Supplementary file 1 [file oncotarget-08-28906-s001.pdf]

Stellettin B induces apoptosis in human chronic myeloid leukemia cells via targeting PI3K and Stat5

SUPPLEMENTARY FIGURES

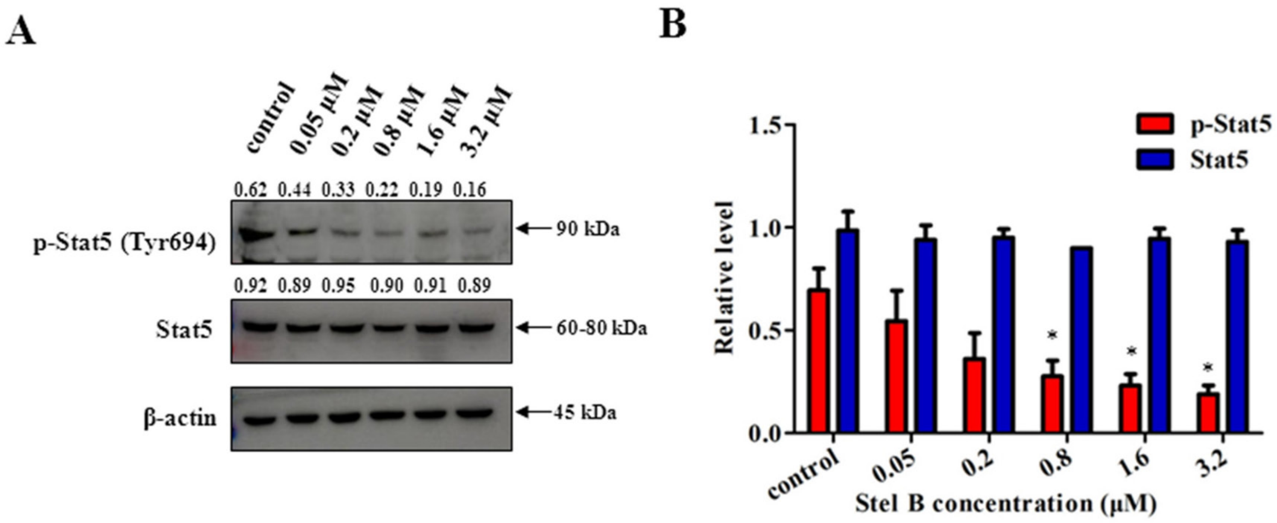

Supplementary Figure 1: Effect of Stel B on Stat5 signaling in other CML KU812 cells. **(A)** After exposure of KU812 cells with increasing doses of Stel B for 48 h, Stat5 and p-Stat5 levels were determined by Western blot. **(B)** Bar graphs represent mean ± SD of three independent experiments. *: $p < 0.05$, compared with the control.

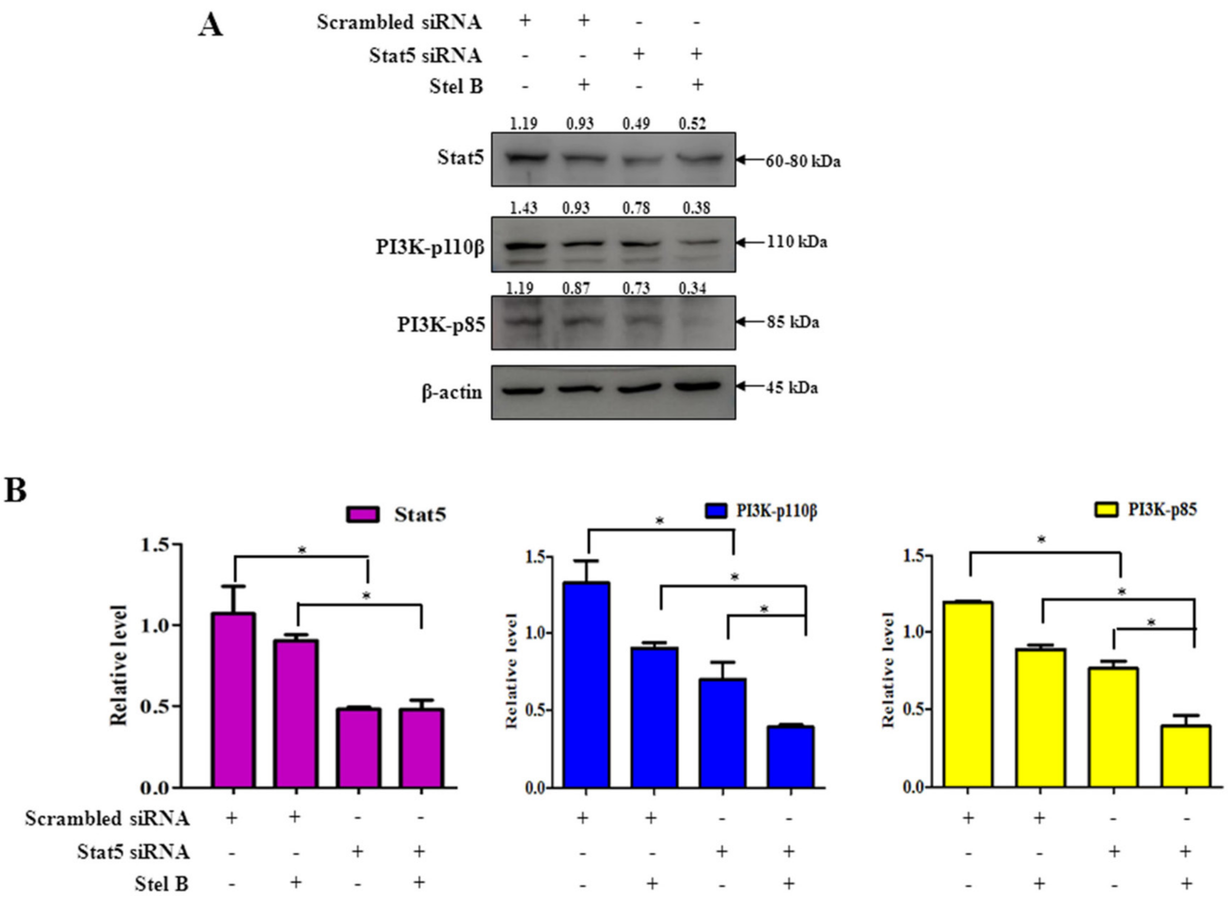

Supplementary Figure 2: Effect of Stat5 siRNA on PI3K expression in K562 cells. **(A)** K562 cells were transfected with siRNA of Stat5A and Stat5B for 6 h, then treated with Stel B (0, 0.036 μM) for 48 h. Expression levels of Stat5, PI3K-p110β and PI3K-p85 were examined by Western blot. **(B)** Bar graphs represent mean ± SD of three independent experiments. *: $p < 0.05$, compared with the control.
